# Electrosurgical devices in gynecologic oncology: a systematic review of clinical studies

**DOI:** 10.3389/fsurg.2026.1772803

**Published:** 2026-03-23

**Authors:** Ekaterina Lazhovska, Kristina Drusany Starič, Katrina Mencin, Marina Jakimovska

**Affiliations:** Division of Gynaecology and Obstetrics, Department of Gynaecology, Faculty of Medicine, University Medical Centre Ljubljana, University of Ljubljana, Ljubljana, Slovenia

**Keywords:** advanced bipolar vessel sealing, electrosurgical devices, gynecological cancer, surgical outcomes, systematic review

## Abstract

**Introduction:**

Electrosurgical instruments are a crucial component in the surgical treatment of gynecologic malignancies, enabling precise dissection and effective hemostasis, influencing blood loss, operative time and complication rates. Considering their importance in oncological outcomes and increasing variety, comparative data in the literature remains limited. Our aim is to comprehensively assess and compare clinical outcomes across electrosurgical modalities in gynecologic cancer surgery.

**Methods:**

Following PRISMA guidelines, we performed a systematic search of three electronic databases (PubMed, Cochrane Library, Scopus). Eligible studies since 2005 enrolled women (≥18 years) undergoing surgery for gynecological neoplasms, in which electrosurgical devices were used. Clinically relevant outcomes, such as intraoperative blood loss, operative time, perioperative complication rates, surgeon's experience and length of hospital stay were compared.

**Results:**

359 publications were reviewed after literature search, 10 of them fitted the inclusion criteria, 1 additional publication was added after references review. Most of the studies (36.4%) evaluated cervical cancer only, followed by studies that included both cervical and endometrial cancer (27.3%), while 18.2% of the studies focused on endometrial and ovarian cancer each. Majority of the articles (82%) evaluated advanced electrosurgical devices alone or in comparison with standard electrosurgery or conventional methods. LigaSure was the most commonly used advanced electrosurgical device, followed by THUNDERBEAT, PlasmaJet™, PlasmaKinetic tissue management system and ERBE BiClamp® forceps. All of the studies assessed more than one perioperative outcome, with the most common being estimated blood loss, operative time and duration of hospitalization.

**Discussion:**

Despite heterogeneous design and outcomes, most findings suggest that advanced electrosurgical devices are associated with mostly better perioperative outcomes in oncological patients undergoing surgery. It can be concluded that the effectiveness of the device and the appropriate instrument selection differs between malignancies, depending on the cancer type and operative procedure performed. Given the diversity of the studies we believe that additional well-designed studies using standardized outcomes sets are needed.

## 1 Introduction

Gynecological malignancies, including cervical, endometrial and ovarian cancer, present one of the leading cancers worldwide, with an incidence of 30.3 per 100 000 women (1). Their management is generally multidisciplinary, including surgery, radiation and chemotherapy; based on tumor type, stage and patient health condition. Despite novel therapeutic interventions, surgery remains the cornerstone of oncology management (2), with procedures ranging from staging to debulking, depending on the stage of the cancer. Surgical outcomes are influenced by surgical skills and appropriate instrument selection, to achieve optimal resection and hemostasis (3). The right choice of a surgical instrument is crucial to minimize blood loss and surrounding tissue injury, enabling the most optimal resection of the tumor to achieve appropriate oncological outcomes, while minimizing postoperative complications. Achievement of hemostasis is a fundamental component that has progressed from conventional mechanical methods, to electrosurgical devices, including diathermy and advanced devices.

Traditional mechanical haemostasis techniques, such as clips have been almost completely abandoned due to cost and possibility of displacement (4). Electrosurgery has become one of the key elements in surgical treatment, used nowadays in more than 80% of surgical procedures, both open and laparoscopic (5). High-frequency electricity in the form of vaporization, desiccation, coagulation and fulguration (6) allows controlled cutting and coagulation at the targeted site (5), with the main advantage being the ability of simultaneous tissue dissection and hemostasis (6). Despite the numerous benefits of electrosurgical devices, serious health hazards have been associated with its use, due to faulty devices or improper use. Complications can include burns of the pads, surgeons' hand or internal organs (5); the reported incidence of the latter is 3.6 per 1,000 laparoscopic procedures and are usually caused by lateral thermal spread. Most of them are recognized postoperatively and can lead to severe morbidity, mortality and increased cost (6).

While conventional diathermy has been the mainstay for surgical practice and it is still widely used, emerging technologies and innovations have led to the evolution of new advanced electrosurgical devices, designed to overcome some of its restrictions (7). These devices operate with less energy and reduce spread of coagulation to adjacent tissues, resulting in decreased risk of undesirable thermal injuries (6), reduced post-operative pain and shorter recovery time (7). Furthermore, their feedback tissue mechanism enables adjusting the power output. Other benefits include the ability of tissue dissection and coagulation using a single instrument, reducing the need for frequent instrument exchanges according to the function of the instrument (6), thereby shortening operative time. This multifunctionality is especially important in minimally invasive surgery, which is becoming increasingly used in gynecological surgery. In minimally invasive surgery factors such as appropriate hemostasis, operative efficiency and limited exchange of instruments are crucial, highlighting the importance of integrated energy devices.

Since the first clinical introduction of electrosurgical instruments by Bovie in the early 19th century to facilitate hemostasis (5), we have seen a continuous progress in types emerging. Despite the versatility and rising numbers of electrosurgical devices on the market and their crucial role in the surgical management of gynecological malignancies, there is a lack of studies comparing their clinical performance. Most existing data had focused mostly on benign conditions. To our knowledge there is no systematic review comparing outcomes of currently available electrosurgical instruments used in oncological gynecology. Our objective is therefore to assess and compare clinical outcomes of different electrosurgical modalities in gynecologic cancer surgery.

## 2 Materials and methods

This systematic review did not require patient consent or Research Ethics Committee approval. Criteria provided by the Preferred Reporting Items for Systematic Reviews and Meta-Analyses (PRISMA) guideline were used to conduct the study.

### 2.1 Search strategy, inclusion and exclusion criteria

A structured search of the three major databases – PubMed, Cochrane Library and Scopus electronic databases was applied to identify relevant articles. They were chosen due to their known comprehensive coverage of the biomedical literature. The search was limited to digital repositories, as we are not aware of any relevant non-electronic sources for this type of research.

Given the rapid advances in oncological surgery, the search period was restricted to studies published within the last 20 years (2005–2025), with the last search conducted on 28.10.2025. Only full-text studies in English that were conducted on adult women (≥18 years) were considered, without restriction on the geographical origin of the publication.

Two reviewers screened the titles and abstracts to identify potentially eligible studies. Full text assessment of the remaining articles was performed, with the final selection completed by KDS and verified by MJ. Duplicates were removed. Any discrepancy regarding eligibility of the studies was resolved through discussion with a third reviewer. Additionally, references of the chosen articles were screened for any other relevant publication.

We included studies that evaluated the use of electrosurgical instruments in patients undergoing surgical treatment for intraabdominal gynecological malignancies. Only studies that evaluated at least one electrosurgical device type were included. It was required that at least one type of relevant surgical outcome was reported, such as intraoperative blood loss, surgical complication, operative time or other clinically significant outcome. Studies were excluded if they evaluated non-oncological surgeries and non-gynecological cancers as well as failed to report outcomes related to the device performance.

For our keyword search strategy we used terms related to electrosurgical devices, endometrial, cervical and ovarian neoplasms as well as gynecologic oncologic procedures. Search strategies were tailored to each database.

Methodological quality of non-randomized studies was calculated using the Newcastle-Ottawa Scale (NOS). Studies having <5 stars are considered low quality, 5-6 moderate and >7 high quality. Case series were addressed using a modified NOS – the *comparability* domain was considered not applicable due to absence of control groups.

Our analysis of electrosurgical devices was structured according to cancer type, rather than type of instrument, in order to provide a diagnosis-specific assessment and comparison.

## 3 Results

### 3.1 Eligible studies

**Figure 1:**
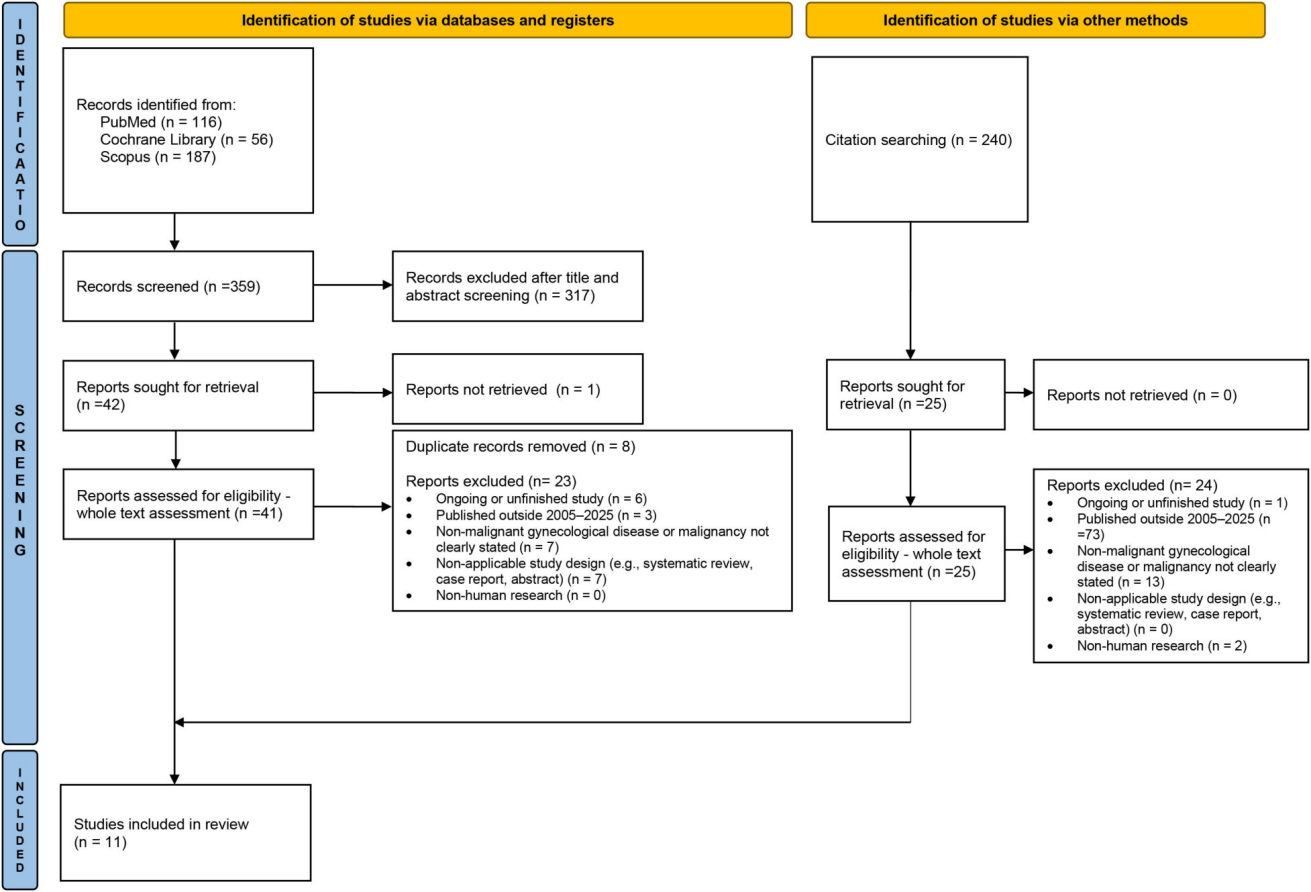
PRISMA flow diagram illustrating the identification, screening, eligibility, and inclusion process for studies via databases, registers, and other methods, resulting in eleven studies included in the review.

The initial literature search yielded a total of 359 records, from which 349 were excluded, because they did not meet the inclusion criteria. A precise manual screening of the reference list of the chosen 10 articles identified 1 additional article, resulting in a total of 11 studies in the final selection. The structured selection process is shown in the PRISMA flow diagram (Figure 1).

### 3.2 Overall analysis

A total of 1,012 female participants were included across 11 studies, published between 2005 and 2021. The number of patients varied between 4 in 391, with a median of 68.

The studies included conventional methods, standard electrosurgery and advanced electrosurgical devices. Instruments from 4 major categories of electrosurgical devices were used, including conventional instruments (54.5%), advanced bipolar systems [LigaSure — different types (27.3%), PlasmaKinetic tissue management system (9.1%), ERBE BiClamp® forceps (9.1%)], neutral plasma energy devices [PlasmaJet™ (18.2%)] and hybrid ultrasonic–bipolar systems [THUNDERBEAT (9.1%)]. Most of the articles (72.7%) included comparison between at least 2 instruments, while the rest were non comparative. Majority of the articles (82%) evaluated advanced electrosurgical devices alone or in comparison with standard electrosurgery or conventional methods.

Regarding the included types of cancer, the leading one was cervical cancer (36.6%), followed by studies that included both endometrial and cervical cancer (27.3%) and ovarian and endometrial cancer contributing 18.2% each. Table 1 summarises relevant study characteristics.

**Table 1:** Key characteristics of the included studies.

Authors, year	Type of study	Number of patients in each group	Type of cancer	Procedure	Devices compared	Outcomes assesed	Limitations
Volcke et al., 2021 (8)	Retrospective cohort study	87, with no control group	Ovarian	Debulking surgery	PlasmaJet™	Operation time, estimated blood loss, duration of hospitalization, perioperative complication rate, resection of all macroscopic disease, relapse and survival rate	No control group; retrospective design; possible selection bias in use of PlasmaJet.
Panuccio et al., 2016 (9)	Retrospective case series	19, with no control group	Ovarian	Debulking surgery	PlasmaJet™	Operation time, estimated blood loss, duration of hospitalization, perioperative complication rate, number of removed lymph nodes, resection of all macroscopic disease, relapse rate	Small sample; no control group; selection bias; limited generalizability.
Lawlor et al., 2015 (10)	Retrospective cohort study	50 versus 100	Endometrial	Robot-assisted hysterectomy and pelvic/para-aortic lymphadenectomy	Electrosurgery with coagulation versus electrosurgery with cutting	Operation time, estimated blood loss, blood transfusion rate and cuff complications	Retrospective design; potential information bias; single-institution robotic practice—limited external validity.
Taşkın et al., 2017 (11)	Prospecitve randomized controlled trial	34 versus 34	Endometrial	Laparoscopic hysterectomy and bilateral salpingo-oophorectomy with retroperitoneal lymphadenectomy	Advanced bipolar instrument (LigaSure Maryland Jaw Laparoscopic 5 mm Sealer) versus conventional surgery	Operation time, estimated blood loss, duration of hospitalization, postoperative pain, number of lymph nodes removed, perioperative complication rate, surgeon’s subjective experience	Single-center randomization; relatively small sample; short-term perioperative outcomes focus.
Li et al., 2012 (12)	Retrospective cohort study	196 versus 195	Cervical	Radical abdominal hysterectomy + pelvic lymph node dissection ± para-aortic lymph node sampling/dissection	ERBE BiClamp® forceps versus conventional suture ligation	Operation time, estimated blood loss, gauze consumption, intraoperative and postoperative blood transfuion rate, hemoglobin decline, closed suction drainage, duration of hospitalization, postoperative complication rate	Retrospective single-center analysis; nonrandomized comparison; potential confounding despite large sample (*n* = 391).
Lee et al., 2007 (13)	Retrospective case-control study	38 versus 38	Cervical	Laparoscopic radical hysterectomy	PlasmaKinetic tissue manegement system versus conventional bipolar electrosurgery (Kleppinger bipolar forceps; Richard Wolf Instruments)	Estimated blood loss, blood transfusion rate, operation time, perioperative complication rate, recurrence rate, duration of hospitalization	Retrospective case–control design with small sample; potential selection bias; limited external validity.
Tamussino et al., 2005 (14)	Prospective nonrandomized controlled trial	31 versus 21	Cervical	Radical abdominal hysterectomy with systematic pelvic +/- paraaortic lymphadenectomy	Bipolar vessel sealing system (LigaSure Vessel Sealing System) versus conventional methods (clamps and suture ligation)	Operation time, blood transfusion rate, number of pelvic nodes removed, febrile morbidity, postoperative stay, postoperative complication rate	Prospective nonrandomized trial; potential underpowering for clinical endpoints; generalizability limited to lymphadenectomy populations.
Shen-Gunther and Shank, 2010 (15)	Retrospective case series	4, with no control group	Cervical	Nerve-sparing radical abdominal hysterectomy with pelvic lymphadenectomy	Ligasure Atlas sealer/divider and Bissenger bipolar shears	Operation time, estimated blood loss, duration of hospitalization, duration until normal postvoid residual volume, perioperative complication rate	Single-surgeon/single-center experience; retrospective design; absence of a control group.
Gallotta et al., 2010 (16)	Prospective randomized controlled pilot trial	30 (one hemipelvis per technique per patient)	Endometrial, cervical	Laparoscopic pelvic lymphadenectomy	Ligaclip (Ethicon Endo-Surgery) versus bipolar coagulation	Lymphoceles after laparoscopic pelvic lymphadenectomy, operation time, estimated blood loss, number of pelvic lymph nodes dissectes, duration of hospitalization	Small pilot sample (*n* = 30); single-center; limited power for complications and long-term outcomes.
Kyo et al., 2009 (17)	Retrospective comparative cohort	18 versus 67	Endometrial, cervical	Radical abdominal hysterectomy with pelvic lymphadenectomy and bilateral adnexectomy	LigaSure Vessel Sealing System with LigaSure Max and LigaSure Precise versus conventional methods	Operation time, estimated blood loss, hemoglobin level reduction, number of pelvic lymph nodes dissected	Nonrandomized, single-center design; small LigaSure subgroup (18/85) with potential selection bias; limited follow-up.
Fagotti et al., 2014 (18)	Prospective randomized controlled trial	25 versus 25	Endometrial, cervical	Laparoscopic radical hysterectomy with bilateral pelvic lymphadenectomy	THUNDERBEAT versus standard electrosurgery (bipolar grasper and cold/monopolar scissors)	Operation time, estimated blood loss, duration of hospitalization, postoperative pain, surgical complication rate	Single-center RCT with modest sample size; powered primarily for operative time, not complications or long-term outcomes.

### 3.3 Outcomes

All of the studies evaluated several perioperative outcomes, with the most common being operative time, intraoperative blood loss and duration of hospital stay. Others included postoperative complications, drain output, formation of lymphoceles, blood transfusion rate, recurrence rate and surgeon's experience. Relevant outcomes are presented in Table 2.

**Table 2:** Summary of clinical outcomes.

Authors, year	Devices compared/used	Operation time (min)	Estimated blood loss (mL)	Duration of hospitalization (days)	Complications
Volcke et al., 2021 (8)	PlasmaJet™	252 (100–550)	700 (200–4000)	10 (4–53)	intraoperative complications: 38%; postoperative complications grade III: 6%; postoperative complications grade IV 3%
Panuccio et al., 2016 (9)	PlasmaJet™	270 (100–420)	700 (100–2,500)	9 (6–21)	intraoperative complications: 32%; postoperative complication grade III: 7%
Lawlor et al., 2015 (10)	Electrosurgery with coagulation versus electrosurgery with cutting	131 vs 129 min; *p* = 0.5	211 vs 228; *p* = 0.29	/	postoperative cuff complications: 4 vs 0; *P* = 0.01
Taşkın et al., 2017 (11)	Advanced bipolar instrument (LigaSure Maryland Jaw Laparoscopic 5 mm Sealer) versus conventional surgery	134.2 ± 29.7 vs 163.5 ± 27.7; *P* < 0.001	176.1 ± 78.2 vs 182.3 ± 104.3; *p* = 0.783	1.9 ± 0.9 vs 2.1 ± 1.1; *p* = 0.480	major complication: 1 (2.9) vs 0; *p* = 0.313
Li et al., 2012 (12)	ERBE BiClamp® forceps versus conventional suture ligation	224.1 ± 36.2 (*P* < 0.001) vs 247.7 ± 47.7	534.8 ± 232.5 vs 769.2 ± 310.4 (*P* < 0.001)	7.1 ± 2.2 vs 8.8 ± 2.5 (*P* < 0.001)	intra-operative blood transfusion: 28.1% vs 75.9 (*P* < 0.001); postoperative blood transfusion rate: 15.6% vs 17.0 (*P* = 0.818); postoperative complications 14.8% vs 23.6% (*P* = 0.027)
Lee et al., 2007 (13)	PlasmaKinetic tissue manegement system versus conventional bipolar electrosurgery (Kleppinger bipolar forceps; Richard Wolf Instruments)	171.8 (65–267) vs 228.9 (121–352), *p* < 0.001	397.4 (100–1,200) vs 564.5 (50–2,400), *p* < 0.05	6.9 (3–16) vs 7.5 (5–15)	Intra-operative complication rectum perforation 1 vs 0; post-operation complications 0 vs 5
Tamussino et al., 2005 (14)	Bipolar vessel sealing system (LigaSure Vessel Sealing System) versus conventional methods (clamps and suture ligation)	199 ± 33 vs 213 ± 45	/	11.4 ± 4.1 vs 11.7 ± 3.5	transfusion rate: 26% vs was 67%
Shen-Gunther and Shank, 2010 (15)	Ligasure Atlas sealer/divider and Bissenger bipolar shears	251 (230–265)	625 (400–900)	5 (4–5)	none
Gallotta et al., 2010 (16)	Ligaclip (Ethicon Endo-Surgery) versus bipolar coagulation	77 (40–122)	47.5 (20–130)	3 (2–11)	incidence of lymphoceles: Ligaclip - 1 (3.3%) vs bipolar coagulation - 9 (30%); *P* = 0.006
Kyo et al., 2009 (17)	LigaSure Vessel Sealing System with LigaSure Max and LigaSure Precise versus conventional methods	242.8 ± 36.1 vs 349.1 ± 82.6; *P* < 0.001	550.9 ± 233.1 vs 745.49.0 ± 230.4; *P* < 0.01	/	pelvic lymph node metastasis: 55.6% vs 22.4%; *P* < 0.05
Fagotti et al., 2014 (18)	THUNDERBEAT versus standard electrosurgery (bipolar grasper and cold/monopolar scissors)	85 (60–160) vs 115 (73–207); *p* = 0.001	50 (20–250) vs 50 (30–500); *p* = 0.52	3 (2–5) vs 3 (2–5); *p* = 0.82	major complications: 0 vs 1 (4.0); *p* = 0.31

### 3.4 Ovarian cancer

Two studies evaluated a total of 106 patients during primary, secondary or tertiary debulking surgery for ovarian cancer using the PlasmaJet™. In the study by Panuccio et al., a complete resection of all macroscopic disease was achieved, while Volcke et al. reports a complete resection in all but one patient. However, the debulking type and FIGO stage of this patient were not reported. The rate of intraoperative complications was 32% and 38%, respectively in each study. Panuccio et al. reported 7% grade III adverse events according to the Clavien-Dindo classification, while Volckle et al. observed 6% grade III and 3% grade IV adverse events. Pneumothorax was observed in 3% of patients across the two studies, with all cases following full-thickness diaphragm resection.

Panuccio et al. observed a recurrence rate of 32% after a median follow-up of 8 months, whereas Volcke et al. reported a substantially higher rate of 82% with a median follow-up of 36 months (8, 9).

### 3.5 Endometrial cancer

Lawlor et al. included 150 patients undergoing robot-assisted hysterectomy and pelvic/para-aortic lymphadenectomy. They compared monopolar electrosurgery using coagulation vs. electrosurgery cutting mode for opening of the vaginal cuff. While no significant difference between the two groups were found for operative time and estimated blood loss, there were more postoperative complications in the coagulation group (4 vs. 0), with the most common being vaginal cuff dehiscence. All of the dehiscences were managed with healing by secondary intention (10).

On the other hand, Taşkın et al. reviewed 68 patients during laparoscopic hysterectomy and bilateral salpingo-oophorectomy with retroperitoneal lymphadenectomy. They compared conventional electrosurgery using Robi forceps with LigaSure. The mean operating time was significantly shorter in the LigaSure group (mean 134.2 min vs. 163.5 min, *p* < 0.001). However, no significant differences were observed for intraoperative blood loss, duration of hospitalization, postoperative pain and in the surgeon's subjective experience (11).

### 3.6 Cervical cancer

Two studies conducted by Shen-Gunther and Shank and Tamussino et al. evaluated the use of LigaSure in radical abdominal hysterectomy with lymphadenectomy, with Shen-Gunther and Shank specifically focusing on nerve-sparing radical hysterectomy. While Tamussino et al. directly compared LigaSure with conventional clamps and suture ligation, Shen-Gunther and Shank did not include a control group; instead, the authors compared the outcomes of their 4 patients with data from previously published studies.

Both studies demonstrated reduced intraoperative blood loss (14, 15). Shen-Gunther and Shank reported that LigaSure use was associated with early recovery of bladder function, with mean time to normal voiding of 17 days, with no long-term neurogenic dysfunction observed. No thermal-related injuries were reported during the follow up. They reported a significantly shorter length of hospital stay, although the operative time was modestly longer (15). On the other hand Tamussino et al. did not observe significant difference in hospital stay or in operating time, possibly because LigaSure was used only for the radical abdominal hysterectomy and not for the lymphadenectomy phase of the surgery (14).

The other two studies assessed pulsed advanced bipolar instruments. Lee et al. reviewed 76 patients undergoing laparoscopic radical hysterectomy comparing PlasmaKinetic system with Kleppinger bipolars forceps. Similarly, Li et al. compared 391 cases of ERBE BiClamps vs. conventional suture ligation in abdominal radical hysterectomy with pelvic node dissection. In the advanced bipolar instrument groups, both studies demonstrated decrease in operative time (median 177 min vs. 232 min, *p* < 0.001 and 224.1 min vs. 247.7 min, *p* < 0.001, accordingly) and intraoperative blood loss (median 350 mL vs. 500 mL, *p* < 0.05 and 534.8 mL vs. 769.2 mL, *p* < 0.001 accordingly). Moreover, there were fewer complications in the advanced electrosurgical devices. Lee et al. reported complication rates of 2.6% vs. 10.4%, while in the study by Li et al. there was a complication rate of 14.8% compared with 23.6% in the control group (12, 13).

Notably, Lee et al. also reported a long-term follow-up period ranging from 12 to 60 months, during which a single recurrence was observed in the conventional method group (13).

### 3.7 Mixed gynecologic malignancies

Three studies focused on both endometrial and cervical malignancies.

The first study by Gallotta et al. evaluated 30 patients undergoing laparoscopic pelvic lymphadenectomy, with each hemipelvis serving as its own control. The efficacy of mechanical ligation with LigaClip vs. bipolar coagulation for the prevention of lymphocele formation was investigated. Closure of lymphatic vessels using Ligaclip had a significantly lower incidence of lymphoceles (3.3% vs. 30%, accordingly) (16).

A second study by Kyo et al. reviewed 85 patients undergoing radical abdominal hysterectomy with pelvic lymphadenectomy, comparing the use of conventional sutures with LigaSure**.** The latter had significantly shorter operative time (mean 242.8 min vs. 349.1 min, *p* < 0.001) and reduced blood loss (mean 583.1 mL vs. 999.0 mL, *p* < 0.005). A significantly higher rate of pelvic lymph node metastasis was reported in the LigaSure group (mean 55.6% vs. 22.4%, *p* < 0.05) (17). A major limitation of the study was the unequal distribution of patients, with only 18 patients in the LigaSure group and 67 in the conventional method group.

In the next study, Fagotti et al. analyzed 50 women undergoing laparoscopic radical hysterectomy with pelvic lymphadenectomy. Standard electrosurgery (bipolar grasper and cold/monopolar scissors) was compared with the THUNDERBEAT device. The use of THUNDERBEAT demonstrated shorter operative time (mean 85 min vs. 115 min, *p* = 0.001) and less postoperative pain, both 8 h and 24 h after surgery. No significant differences were observed in other perioperative outcomes such as estimated blood loss and duration of hospital stay (18).

### 3.8 Study quality and limitations

Several advantages of the included studies should be acknowledged. Most of them had comparable study designs and assessed a wide range of relevant intraoperative and postoperative outcomes, with some of them having a big number of patients, up to 391. Notably, many of the studies employed a prospective study design.

Limitations of each study are shown in Table 1, with the most common being small sample size, short-term follow-up and nonrandomized study designs. The majority of the studies focused primarily on perioperative parameters, only a few of them assessed oncologic outcomes. The surgeon's experience with using different devices was rarely evaluated.

Regarding methodological quality, four studies showed moderate (NOS scores 6–7), while four revealed low quality (NOS score of 3), mainly due to not having a control group. Results are summarized in Table 3. The three randomized trials were not assessed with a NOS; they had appropriate randomization process, however there was no blinding of surgeons.

**Table 3:** Methodological quality of studies using the Newcastle-Ottawa scale.

Authors, year	Selection	Comparability	Outcome	Score
	Max 4 Stars	Max 2 Stars	Max 3 Stars	Max 9 Stars
Volcke et al., 2021 (8)	★★	/	★	3
Panuccio et al., 2016 (9)	★★	/	★	3
Lawlor et al., 2015 (10)	★★★	★	★★	6
Taşkın et al., 2017 (11)	not applicable (randomized study)			
Li et al., 2012 (12)	★★★★	★	★★	7
Lee et al., 2007 (13)	★★★	★	★★	6
Tamussino et al., 2005 (14)	★★★	★	★★	6
Shen-Gunther and Shank, 2010 (15)	★★	/	★	3
Gallotta et al., 2010 (16)	Not applicable (randomized study)			
Kyo et al., 2009 (17)	★★★★	★	★★	6
Fagotti et al., 2014 (18)	Not applicable (randomized study)			

## 4 Discussion

Electrosurgical devices are crucial in the surgical treatment of gynecological malignancies, significantly affecting operative efficiency and postoperative outcomes. In this systematic review we evaluated studies in which electrosurgical devices were used during gynecologic oncological surgeries and compared clinically relevant outcomes.

### 4.1 Ovarian cancer

Both studies by Panuccio et al. and Volcke et al. have shown that the use of PlasmaJet is safe and efficient during debulking surgery for ovarian cancer, accomplishing complete macroscopic resection in 99% of patients. This is likely attributable to the device's design, which allows for superficial tissue cutting with minimal thermal spread. These characteristics are advantageous in the treatment of peritoneal lesions, commonly present in advanced stages of ovarian cancer, in which complete resection of all macroscopic lesions is a crucial prognostic factor. A major limitation of both studies is the absence of a control group, as PlasmaJet was used in all enrolled patients. Even though the device has a mechanism of limiting depth of penetration, caution is warranted during full-thickness diaphragm resections due to potential risk of iatrogenic pneumothorax. High disease recurrence rates were observed in both studies; however, the majority of included patients (83%) were diagnosed in advanced stages of the disease (FIGO III or IV) (8, 9). However, the use of PlasmaJet did not influence the recurrence rate.

### 4.2 Endometrial cancer

The standard of care for endometrial cancer treatment is hysterectomy, usually performed using a minimally invasive technique. Vaginal cuff complications, such as necrosis, dehiscence and bowel evisceration, are an established postoperative risk. Lawlor et al. compared vaginal cuff complications in robotic assisted surgery using monopolar electrosurgery in coagulation mode vs. cutting mode. Whereas there were no significant differences for operating time and estimated blood loss, all of the reported complications occurred in the coagulation mode group (10). This could be due to excessive thermal spread that can impair tissue perfusion and weaken healing; additionally, malignancy itself is a well-recognized independent risk factor for cuff dehiscence (19). An important limitation of this study is the uneven distribution between patients (1:2).

In a subsequent study conducted by Taşkın et al., conventional bipolar electrosurgery was compared with LigaSure in laparoscopic hysterectomy and bilateral salpingo-oophorectomy with retroperitoneal lymphadenectomy. The only perioperative outcome that differed between groups was the notably shorter operating time by approximately 29.3 min in the LigaSure group (11). Bipolar instruments require more time for coagulation, can not cut tissue and hence require another instrument to cut (4), which could be the reason for the longer operative time.

The use of LigaSure is generally associated with better hemostasis due to its high coaptive pressure and energy, which forms a permanent plastic-like seal of large vessels (7). In contrast, bipolar diathermy provides less tissue coaptation, which might lead to incomplete coagulation and haemostasis (6). Additionally, the jaws of the bipolar instruments can potentially stick to the tissue, making it hard to remove after coagulation (20) and the adherence may cause tearing of adjacent vessels (4). Surgeons have to rely on visual changes such as vapour and color changes to assess effectiveness of coagulation while using diathermy (6). However, there was no significant difference in blood loss between the groups (11). Similarly, studies including laparoscopic hysterectomies for benign conditions did not demonstrate a reduction in intraoperative blood loss with the use of LigaSure in comparison with bipolar electrosurgery (21, 22). On the other hand, studies including benign and malignant conditions in other fields, such as abdominal, urological and thyroid surgeries, have shown reliable hemostasis and reduced intraoperative blood loss with LigaSure (23–25). This might suggest that gynecologic cancers have distinct biological potential, with unique anatomic and procedural characteristics that may influence the benefits of different vessel sealing technologies.

Even though there were no differences in surgeon's experience in both modalities, the authors note that advanced instruments may reduce anxiety for thermal injuries in less experienced surgeons (11). Although advanced bipolar devices could shorten operating time, conventional methods could be equally effective. Based on the advantages LigaSure offers, its higher cost should be weighed against the modest clinical benefits observed.

### 4.3 Cervical cancer

Despite the declining mortality due to screening programs, cervical cancer is still an important global health challenge, with surgery being crucial for treatment (12).

Tamussino et al. and Shen-Gunther and Shank demonstrated that the use of LigaSure for cervical cancer reduces intraoperative blood loss (14, 15). However, in the second study the operative time was modestly longer, which could be attributed to the thorough dissection needed for nerve-sparing techniques (15). The absence of electrothermal complications in this study (15) could be associated with the minimal lateral thermal spread associated with LigaSure, ranging from 0.5 mm to 2 mm (26), compared with 1–6 mm for conventional bipolar forceps (17). Additionally, LigaSure delivers the minimum energy needed for effective cauterisation, discontinuing it when the seal cycle is completed (7). However, since there were only 4 patients, these findings need to be interpreted with caution.

Two studies evaluated the impact of advanced pulsed bipolar systems, PlasmaKinetic and ERBE BiClamp, during radical hysterectomy. Both studies by Lee et al. and Li et al. demonstrated decreased intraoperative blood loss, operative time and complications in the advanced pulsed bipolar group (12, 13). Pulsed bipolar system is a subgroup of the advanced bipolar instruments that delivers intermittent pulses, instead of a continuous stream of energy, achieving hemostasis while allowing the tissue to cool down between pulses, thereby additionally reducing thermal injuries (20), which is probably the reason for the better perioperative outcomes.

Although no urine fistulas were found in the BiClamp group, the authors warn that the ureter tunnel should be opened with a cold knife to avoid thermal injury, since the BiClamp forceps can cause 1–3 mm collateral thermal injuries. Thus, particular care is warranted around other vulnerable structures such as vessels and nerves (12). A rectal perforation was the only complication in the PlasmaKinetic group, which occurred during the right utero-sacral ligament dissection. The authors state that it most probably happened due to disease status rather than device-related factors (13).

PlasmaKinetic cutting forceps may be difficult to use in dissection of larger flat areas (for example in colpotomy) due to difficulty in grasping. In such cases, unipolar scissors or the PlasmaKinetic needle electrode can be used (13).

### 4.4 Mixed gynecologic malignancies

Pelvic lymphadenectomy is an important step in the staging and treatment of several gynecologic cancers. However, lymph node dissection and inadequate afferent lymphatic vessels closure can result in a formation of lymphocele resulting in pelvic pain, unilateral leg edema, hydronephrosis and thromboembolic events. Gallota et al. evaluated the efficacy of mechanical ligation with LigaClip vs. bipolar coagulation for the prevention of lymphocele formation in patients with endometrial and cervical cancer. They demonstrated significantly lower incidence of lymphoceles with mechanical ligation, indicating that use of LigaClip to clamp lymphatic vessels may prevent formation of lymphoceles. It could be more successful since it provides a physical, more reliable closure, whereas bipolar coagulation could lead to leaking if incompletely sealed due to the inability of lymphatic vessels to constrict and weak clotting characteristics (16).

In a study by Kyo et al., the use of LigaSure was associated with a significantly shorter operative time and reduced blood loss in comparison with conventional sutures in patients undergoing radical abdominal hysterectomy. This could be due to the device's ability to achieve reliable hemostasis during resection of critical structures, such as the uterosacral ligaments (17), by effectively sealing vessels from 1 to 7 mm in diameter with a permanent seal that can tolerate three times the normal systolic pressure (4). A major limitation of the study was the unequal distribution between groups.

In a subsequent study by Fagotti et al., the THUNDERBEAT device was compared with standard electrosurgery during laparoscopic radical hysterectomy with pelvic lymphadenectomy for endometrial and cervical cancer. The THUNDERBEAT has the benefits of both ultrasonic and advanced bipolar energy integrated in a single instrument, which allows for tissue dissection with ultrasonic and reliable vessel sealing using bipolar energy. THUNDERBEAT demonstrated reduction in operative time, by approximately 30 min, even in more complicated cases, such as in overweight patients. Operative time is an important factor, which the authors report as the probable reason for the less postoperative pain in the THUNDERBEAT group (18).

Allthough Gallotta et al. demonstrated reduced incidence of lymphoceles with the use of mechanical ligation (LigaClip), this did not lead to a change in surgical standards within the same research group, as in a later study by Fagotti et al. standard electrosurgery remained the reference technique. This highlights that at the core, surgical principles in gynecologic oncology are defined by established guidelines and remain consistent. While the instruments and energy platforms continue to evolve, technological innovations represent refinements of technique rather than changes in surgical standards.

### 4.5 Conclusion

The presented evidence suggests that the use of advanced electrosurgical instruments devices is generally associated with improvement of perioperative outcomes in oncological gynecological surgery with results varying between different types of cancers and operations. These findings highlight the importance of procedure and cancer specific nature of energy device performance.

Although a subgroup analysis based on tumor type was performed, there is a substantial heterogeneity in study designs that needs to be taken in consideration when interpreting this systematic review analysis. The overall quality of the included studies was moderate.

Despite the widespread use of electrosurgical devices in gynecological oncology, comparative data remains insufficient. Studies usually evaluate devices in broad categories (e.g., “diathermy” vs. “advanced energy devices”), leading to a notable lack of studies comparing advanced devices among themselves (e.g., THUNDERBEAT vs. Harmonic scalpel). This gap in evidence is clinically relevant, since choice of energy device may influence intraoperative blood loss, operative time, injuries of surroundings tissues, potentially further increasing morbidity and delaying adjuvant therapy. Perioperative effect may extend to long-term outcomes in terms of life quality, psychosexual and social well-being. Therefore, additional research including well-designed randomized controlled trials evaluating the use of different electrosurgical modalities for specific types of gynecological cancers is needed, using standardized perioperative sets.

## Data Availability

The original contributions presented in the study are included in the article/supplementary material, further inquiries can be directed to the corresponding author.
